# Paucibacillary leprosy presenting as fibular neuropathy: A case of autochthonous zoonotic exposure in endemic Florida

**DOI:** 10.1371/journal.pntd.0014456

**Published:** 2026-06-22

**Authors:** Kyle Ruffing

**Affiliations:** Department of Neurology, University of Florida, Gainesville, Florida, United States of America; University of Bremen: Universitat Bremen, GERMANY

## Abstract

We report a case of paucibacillary leprosy presenting as fibular neuropathy in a patient from Florida, an area with documented autochthonous transmission of Mycobacterium leprae. The patient exhibited neuropathic symptoms and a characteristic skin lesion, ultimately confirmed by biopsy with histopathological findings consistent with paucibacillary Hansen’s disease, with negative Fite-Faraco staining and negative M. leprae PCR. The patient reported no travel history to internationally endemic areas; zoonotic exposure through frequent contact with armadillo-inhabited soil was identified as the most probable source of infection. This case underscores the importance of thorough physical examination and consideration of autochthonous zoonotic exposure in regions with documented environmental transmission.

## Author summary

Leprosy remains a neglected tropical disease globally, including the United States, particularly in the Southeast where autochthonous zoonotic transmission from armadillos is documented. This case illustrates diagnostic challenges and emphasizes vigilance for infectious etiologies in neuropathic presentations, particularly when travel history is absent but local environmental exposure to armadillos is present.

## Introduction

Leprosy (Hansen’s disease) is a chronic infection caused by *Mycobacterium leprae*, affecting skin and peripheral nerves. It is classified as a neglected tropical disease (NTD) globally, with an estimated 200,000 new cases reported worldwide each year. In the United States, leprosy is uncommon, with approximately 200 cases diagnosed annually over the past decade. The majority occur in individuals who have immigrated from internationally endemic countries; however, roughly a quarter of cases are diagnosed in U.S.-born individuals with no known contact with a leprosy patient, likely infected through environmental exposure to *M. leprae*, most commonly through contact with armadillos, as 10–20% of armadillos in the southern states are infected with *M. leprae*. Notably, leprosy cases in Florida have nearly doubled in the past year according to the Florida Department of Health [1], with 31 cases reported in 2025 compared to 16 reported in 2024. These autochthonous cases in Florida are most frequently linked to armadillo exposure. Clinical examination and a high index of suspicion are required for early diagnosis. Electrodiagnostic studies can aid in identifying neuropathic involvement. We present a case of paucibacillary leprosy manifesting as fibular neuropathy.

### Ethics statement

This work did not require review or approval by an Institutional Review Board or Ethics Committee, as it represents a case report of a single patient and does not constitute human subjects research as defined by applicable regulatory guidelines. Written informed consent was obtained from the patient for publication of this case report and all accompanying images, including clinical photographs and histopathological figures.

### Case presentation

A 55-year-old woman with no travel history outside the United States presented with progressive burning pain between her first and second toes, accompanied by numbness and mild weakness. One year before presentation, she developed progressive burning pain between her first and second toes with numbness of the lateral portion of the first toe and medial portion of the second toe. The pain was exacerbated by standing but unrelieved by a mobility boot. She was thought to have a lumbar radiculopathy despite the absence of radicular features, and symptoms were unrelieved by lumbar epidural injection. Symptoms persisted despite lumbar interventions. In total, 18 months elapsed from initial symptom onset to confirmed diagnosis of leprosy, illustrating the diagnostic delays common in this disease. At the same time, she developed a rash on the posterior thigh. Evaluation by dermatology one year ago revealed a coin-shaped plaque with an erythematous border and central yellow clearing, and was thought to be pigmented purpuric dermatitis, though multiple topical treatments were ineffective.

At the time of her EMG, her strength was noted to be 5/5, with 2 + reflexes, and diminished pinprick sensation in the first webspace. Her nerve conduction studies and needle electromyography results are shown in Table 1.

**Table 1:** Motor nerve conduction studies.

Nerve	Side	Site	Latency (ms)	Normal Latency	Amplitude (mV)	Normal Amplitude	Velocity (m/s)	Normal Velocity
Fibular (EDB)	Right	Ankle	4.7	<6.5	0.28	>2.0		
Fibular (EDB)	Right	Fibular Head	11.5		0.2	>2.0	46	>42
Fibular (EDB)	Right	Popliteal Fossa	13.6		0.2		48	>42
Tibial (AHB)	Right	Ankle	3.5	<6.1	5.4	>5.3		
Fibular (EDB)	Left	Ankle	4.0	<6.5	3.0	>2.0		
Fibular (EDB)	Left	Fibular Head	9.4		2.4		54	>42
Fibular (EDB)	Left	Popliteal Fossa	11.1		2.4		53	>42
Tibial (AHB)	Left	Ankle	4.1	<6.1	4.9	>5.3		
**Sensory Nerve Conduction Studies**								
Nerve	Side	Site	Peak Lat (ms)	Normal Latency	Amplitude (µV)	Normal Amplitude	Velocity (m/s)	Normal Velocity
Fibular Sensory	Right	Lateral Leg	3.6	<4.2	17	>5	47	>39
Sural	Right	Posterior Leg	3.6	<4.5	12	>4	50	>39
Fibular Sensory	Left	Lateral Leg	2.7	<4.2	20	>5	52	>39
Sural	Left	Posterior Leg	2.7	<4.5	13	>4	52	>39
**Needle Electromyography**								
Muscle	Side	Insertional Activity	Spontaneous Activity	MUAP Amplitude/Duration	Poly	Recruitment	Comments	
Tibialis Anterior	Right	Increased	2 + fibrillations	Increased/Normal	0	Nml		
Gastrocnemius	Right	Nml	Nml	Nml/Nml	0	Nml		
Extensor Digitorum Brevis	Right	Increased	2 + fibrillations	Increased/Nml	0	Reduced	One motor unit	
Fibularis Longus	Right	Nml	Nml	Nml/Nml	0	Nml		
Extensor Digitorum Brevis	Left	Nml	Nml	Nml/Nml	0	Nml		

Table 1 displays motor and sensory nerve conduction data and needle electromyography data. Notably, the right fibular motor response was low amplitude with normal sensory responses consistent with a deep fibular mononeuropathy. Needle EMG of the right Extensor Digitorum Brevis (EDB) and Tibialis Anterior demonstrated increased insertional activity and fibrillation potentials, indicating active denervation in the deep fibular nerve distribution. These electrodiagnostic findings are consistent with a right Deep Fibular mononeuropathy and supported the localization of nerve injury, prompting further evaluation for an infectious etiology. Abbreviations: EDB-Extensor Digitorum Brevis, AHB-Abductor Hallucis Brevis, mv-millivolts, m/s-meters/second, µv-microvolts, MUAP-motor unit action potential, Nml-normal.

**Fig 1:**
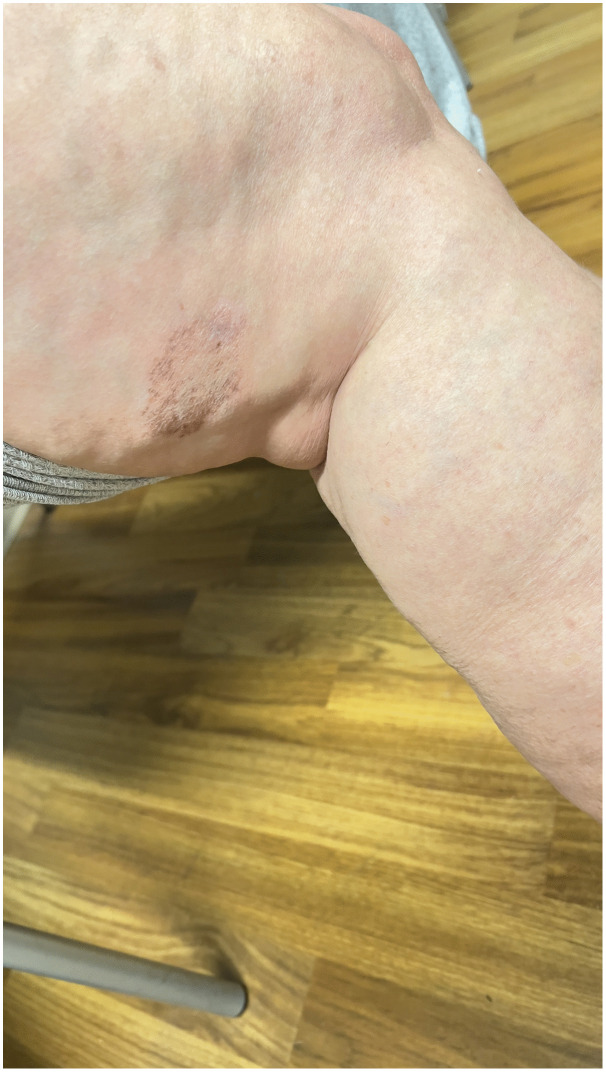
Skin lesion, right posterior thigh.

While performing fibular nerve testing across the knee, the patient’s gown was moved above the thigh, and the postero-lateral skin lesion was noted (Fig 1). The electromyographer used a safety pin to test sensation around the lesion and found it normal, but pinprick in the center of the lesion was felt to be absent when tested. Skin biopsy was performed and the specimen was sent to the National Hansen’s Disease Program (NHDP) for evaluation. Histopathological examination (H&E staining) revealed granulomatous inflammation with lymphocytic infiltration of the dermis, consistent with leprosy. Fite-Faraco staining was negative for acid-fast bacilli. *M. leprae* PCR was negative. Based on the WHO classification of one skin lesion with confirmed histopathology, the patient was classified as paucibacillary leprosy.

The patient reports no travel outside the United States and no known exposure to patients with Hansen’s Disease. The patient has resided in Florida for 25 years. She reports frequent soil exposure in her yard, with frequent armadillo sightings burrowing under her house for many years, preceding the onset of her neuropathic symptoms by at least two years. She recalls being waist-deep in mud at times in her yard and has attempted unsuccessfully to trap armadillos. She has frequent contact with the soil in this rural setting, filling in the holes dug by these armadillos. This prolonged environmental exposure to potentially *M. leprae*-contaminated soil and armadillos represents the most plausible route of autochthonous transmission, consistent with the long and variable incubation period of leprosy (typically 2–12 years).

Treatment with multidrug therapy (MDT) was initiated per WHO and NHDP guidelines for paucibacillary disease, consisting of rifampin, moxifloxacin, minocycline and Vitamin D for 12 months with improvement of her right thigh skin lesion.

## Discussion

Leprosy incidence in the U.S. is increasing dramatically in the Southeast, likely associated with autochthonous zoonotic transmission from the 9-banded armadillo [2,3,4]. Genetic confirmation of mycobacterial strains is consistent with regional autochthonous zoonotic transmission. A particularly relevant cluster of 15 human and 10 armadillo cases of zoonotic leprosy was identified in central Florida, with genotyping revealing strain 3I-2-v15; the patient described in this report likely carries the same genotype [4]. Armadillos are a unique host for this obligate intracellular parasite. Up to 20% of armadillos are infected [5]; the entire animal may harbor trillions of *M. leprae* bacilli, though tissue concentrations are typically in the range of 10⁸ to 10⁹ bacilli per gram of tissue (one hundred million to one billion). Their lower body temperature makes them an ideal host.

This case further illustrates that autochthonous transmission can occur without any travel to internationally endemic regions, highlighting the importance of eliciting a detailed local environmental and zoonotic exposure history, including duration and timing relative to symptom onset, in patients presenting with peripheral nerve and skin lesion in Florida and the southeastern United States. Delay in diagnosis is common, leading to increased disability. In this case, approximately 18 months elapsed before the correct diagnosis was established, during which the patient underwent inappropriate spinal interventions. Clinical manifestations vary from skin lesions to neuropathy. Electrodiagnostic studies may assist in monitoring nerve involvement and treatment toxicity. This case highlights the importance of thorough physical examination and consideration of autochthonous infectious causes in neuropathic presentations.

## Conclusions

Clinicians should maintain a high index of suspicion for leprosy as a globally relevant neglected tropical disease, in autochthonous areas such as Florida, especially when neuropathic symptoms coexist with skin lesions and a history of local zoonotic exposure, even in the absence of international travel. Increased awareness may lead to earlier treatment and improved outcomes.
