# Supplementary material for: Paucibacillary leprosy presenting as fibular neuropathy: A case of autochthonous zoonotic exposure in endemic Florida
Source: PLoS Negl Trop Dis. 2026 Jun 22;20(6):e0014456. doi: 10.1371/journal.pntd.0014456 (PMC13313367; doi:10.1371/journal.pntd.0014456)
Supplement: Attachment — Submitted filename: Response to Reviewers.docx [file pntd.0014456.s002.docx]

**Date:** May 19, 2026

**Journal:** PLOS Neglected Tropical Diseases

**Manuscript Number:** PNTD-D-26-00031

**Title:** Paucibacillary Leprosy Presenting as Fibular Neuropathy: A Case of Autochthonous Zoonotic Exposure in Endemic Florida

**Authors:** Ruffing et al.

Dear Editors and Reviewers,

We are grateful to the editors and all three reviewers for their careful reading of our manuscript and their constructive, detailed feedback. We believe these revisions have substantially strengthened the manuscript. Below, we provide a point-by-point response to each comment. All changes are reflected in the tracked-changes version of the revised manuscript.

**Response to Reviewer #1**

**Comment 1:** *"Include 'travel history to endemic areas and zoonotic exposure' in the abstract and discussion."*

**Response:** Thank you for this suggestion. We have revised the abstract to explicitly state that the patient had no travel history to internationally endemic areas and that zoonotic exposure through contact with armadillo-inhabited soil was identified as the most probable source of infection. The discussion has likewise been expanded to emphasize the importance of eliciting detailed local zoonotic and environmental exposure histories, including the absence of international travel, in patients presenting with peripheral neuropathy in Florida and the southeastern United States. PNTD-D-26-00031.pdf

**Comment 2:** *"Leprosy is an NTD globally, not just in the USA."*

**Response:** We agree and apologize for this imprecision. The Author Summary has been revised to read: "Leprosy remains a neglected tropical disease **globally**, including in the United States, particularly in the Southeast where autochthonous zoonotic transmission from armadillos is documented." PNTD-D-26-00031.pdf

**Comment 3:** *"Based on your assessment this case is 'autochthonous', not merely endemic. Much better term."*

**Response:** We thank the reviewer for this important terminological clarification. "Autochthonous" is indeed the more precise and scientifically appropriate term, as it specifies that transmission occurred locally within the United States rather than through travel to an internationally endemic region. We have replaced all instances of "endemic" (when used to describe this patient's transmission) with "autochthonous" throughout the manuscript, including the title, abstract, introduction, discussion, and conclusions. PNTD-D-26-00031.pdf

**Comment 4:** *"Armadillos may harbor 'trillions of leprosy bacilli' in the entire animal, but only 'billions of bacilli per gram.'"*

**Response:** Thank you for this factual correction. The relevant sentence in the Discussion has been revised to: "Up to 20% of armadillos are infected; the entire animal may harbor trillions of *M. leprae* bacilli, though tissue concentrations are typically in the range of 10⁸ to 10⁹ bacilli per gram of tissue (one hundred million to one billion)." PNTD-D-26-00031.pdf

**Response to Reviewer #2**

**Comment 1:** *"The authors state that the patient had 'paucibacillary leprosy confirmed by biopsy,' but no details are provided on what was actually done on the tissue... Was acid-fast bacilli staining (e.g. Fite-Faraco) performed... Was M. leprae PCR carried out... What were the histopathological findings? It is also unclear whether the sample was sent to the National Hansen's Disease Program (NHDP)."*

**Response:** This is a critical and valid concern, and we thank the reviewer for identifying this gap. We have substantially expanded the diagnostic section of the Case Presentation. The revised manuscript now states that the biopsy specimen was sent to the National Hansen's Disease Program (NHDP) for evaluation. Histopathological findings on H&E staining, Fite-Faraco staining results, and *M. leprae* PCR results are now explicitly reported.

**Comment 2:** *"The exposure history provided for the patient remains vague and poorly contextualized... no temporal or quantitative information is provided... no information on whether exposure preceded symptom onset."*

**Response:** We agree that this is a significant weakness and have substantially revised the exposure history in the Case Presentation. The revised text now includes the duration of the patient's Florida residence, the approximate timeline of armadillo and soil exposure relative to symptom onset, and an explicit acknowledgment that the incubation period of leprosy (typically 2–12 years) is consistent with the patient's exposure history. We acknowledge that the absence of more granular quantitative exposure data is a limitation of this case report and have noted this in the Discussion.

**Comment 3:** *"The manuscript requires major revision. Key diagnostic details are missing, the writing lacks clarity and precision, and the conclusions regarding zoonotic exposure are not adequately supported."*

**Response:** We have undertaken a thorough revision of the entire manuscript with attention to clarity, precision, and scientific rigor. Diagnostic details have been expanded as described above, the zoonotic narrative has been reframed with appropriate caution where data are limited, and the conclusions have been revised to reflect the available evidence more accurately.

**Response to Reviewer #3**

**Comment 1:** *"This statement could really be improved by providing some real numbers... Please provide the number of cases of leprosy diagnosed in Florida in 2025 for context... Please cite the web page for the Florida Department of Health."*

**Response:** We thank the reviewer for this constructive suggestion. The Introduction has been substantially revised to include quantitative context: approximately 200 cases are diagnosed annually in the United States; roughly a quarter occur in U.S.-born individuals without known patient contact, most likely infected through environmental or zoonotic exposure; and 10–20% of armadillos in southern states carry *M. leprae*. The Florida Department of Health webpage has been added as a citation, and the 2025 Florida case count has been inserted.

**Comment 2:** *"The more relevant citation of the cluster of zoonotic leprosy... in central Florida... should be included: Sharma R et al. 2015. Zoonotic leprosy in the Southeastern United States. Emerg Inf Dis 21: 2127-2134."*

**Response:** We thank the reviewer for directing us to this highly relevant reference. The Sharma et al. (2015) citation has been added to both the Introduction and Discussion. We also note the reviewer's suggestion that the patient likely carries the same genotype (3I-2-v15) identified in that cluster.

**Comment 3:** *"How many months exactly passed from the initial symptoms until leprosy was diagnosed, seems like almost 2 years."*

**Response:** We agree that documenting the diagnostic delay is important both clinically and for public health messaging. The revised Case Presentation now explicitly states the duration from initial symptom onset to confirmed diagnosis. We have also added discussion of the consequences of delayed diagnosis, including unnecessary lumbar interventions and progressive nerve damage.

**Comment 4:** *"'Her nerve conduction studies and needle testing are shown (Table 1).' Table 1 does not seem to exist."*

**Response:** We sincerely apologize for this oversight. Table 1, containing the motor and sensory nerve conduction study results as well as the needle EMG findings, has been reattached and properly referenced in the manuscript. We confirm it was included in the original submission files and have ensured it is correctly linked in this revision.

**Comment 5:** *"A figure of the stained (H&E) skin section showing lymphocytic infiltration characteristic of granulomatous disease... Was there also staining of a section with Fite-Faraco to determine if acid-fast bacilli were present?"*

**Response:** We confirm that the specimen was sent to the National Hansen’s Disease Program (NHDP) for evaluation. Fite-Faraco staining was negative for acid-fast bacilli, which is not uncommon in paucibacillary leprosy. M. leprae PCR was negative as noted now in the case report.

**Comment 6:** *"Please be more specific [about treatment]. If this individual was classified with paucibacillary disease, standard treatment with MDT is usually for 6 months with rifampicin, dapsone and clofazimine."*

**Response:** Thank you for prompting greater specificity. The treatment section has been revised to state: "Treatment with multidrug therapy (MDT) was initiated per WHO and NHDP guidelines for paucibacillary disease, consisting of rifampicin, moxifloxacin, minocycline, and Vitamin D for a total duration of 6 months."

**Comment 7:** *"'armadillos are infected with trillions of bacilli per gram of tissue'... it is more likely in the range of between 1E8 to 1E9... One trillion bacilli would be 1E12, not possible."*

**Response:** We thank the reviewer for this important factual correction. As also noted by Reviewer #1, the text has been revised to accurately reflect that tissue concentrations are in the range of 10⁸ to 10⁹ bacilli per gram, while acknowledging that the entire animal may harbor trillions of bacilli in aggregate.

**Comment 8:** *"The two Tables showing Motor Nerve Results and Sensory Nerve Results show numbers in boxes that are colored green (normal range?) or red (abnormal range?)... Please define abbreviations EDB, Ins Act, Fibs, Psw... Please explain what these values indicate and how they led to or confirmed the diagnosis of leprosy."*

**Response:** We have added a detailed explanation of the electrodiagnostic tables in the Case Presentation. Color coding is now defined (green = within normal reference limits per AANEM standards; red = outside normal limits). All abbreviations have been defined: EDB (extensor digitorum brevis), Ins Act (insertional activity), Fibs (fibrillation potentials), and PSW (positive sharp waves). The clinical significance of these findings — specifically, active denervation in the fibular nerve distribution consistent with axonal fibular neuropathy — is now explained, along with how these findings directed the diagnostic workup toward an infectious etiology.

We thank the reviewers again for their thorough and constructive engagement with this manuscript. We believe the revised manuscript is substantially improved and hope it is now suitable for publication in PLOS Neglected Tropical Diseases.

Sincerely,

**Kyle William Ruffing, MD**
Assistant Professor; Director, Neuromuscular Fellowship Program
University of Florida College of Medicine
Gainesville, FL, USA
