# Supplementary material for: Paucibacillary leprosy presenting as fibular neuropathy: A case of autochthonous zoonotic exposure in endemic Florida
Source: PLoS Negl Trop Dis. 2026 Jun 22;20(6):e0014456. doi: 10.1371/journal.pntd.0014456 (PMC13313367; doi:10.1371/journal.pntd.0014456)
Supplement: Peer review history

# Peer review: Decision Letter 0

**DOI:** 10.1371/journal.pntd.0014456.r001

**Authors:** Mathieu Picardeau, Anil Fastenau

16 Feb 2026

PNTD-D-26-00031

Paucibacillary Leprosy Presenting as Fibular Neuropathy: A Case of Zoonotic Exposure in Endemic Florida

PLOS Neglected Tropical Diseases

Dear Dr. Ruffing,

Thank you for submitting your manuscript to PLOS Neglected Tropical Diseases. After careful consideration, we feel that it has merit but does not fully meet PLOS Neglected Tropical Diseases's publication criteria as it currently stands. Therefore, we invite you to submit a revised version of the manuscript that addresses the points raised during the review process.

Please submit your revised manuscript within by Apr 17 2026 11:59PM. If you will need more time than this to complete your revisions, please reply to this message or contact the journal office at plosntds@plos.org. When you're ready to submit your revision, log on to https://www.editorialmanager.com/pntd/ and select the 'Submissions Needing Revision' folder to locate your manuscript file.

Please include the following items when submitting your revised manuscript:

* A letter that responds to each point raised by the editor and reviewer(s). You should upload this letter as a separate file labeled 'Response to Reviewers'. This file does not need to include responses to any formatting updates and technical items listed in the 'Journal Requirements' section below.

* A marked-up copy of your manuscript that highlights changes made to the original version. You should upload this as a separate file labeled 'Revised Manuscript with Track Changes'.

* An unmarked version of your revised paper without tracked changes. You should upload this as a separate file labeled 'Manuscript'.

If you would like to make changes to your financial disclosure, competing interests statement, or data availability statement, please make these updates within the submission form at the time of resubmission. Guidelines for resubmitting your figure files are available below the reviewer comments at the end of this letter.

We look forward to receiving your revised manuscript.

Kind regards,

Anil Fastenau, M.D., M.Sc.

Guest Editor

PLOS Neglected Tropical Diseases

Mathieu Picardeau

Section Editor

PLOS Neglected Tropical Diseases

Shaden Kamhawi

co-Editor-in-Chief

PLOS Neglected Tropical Diseases

orcid.org/0000-0003-4304-636XX

Paul Brindley

co-Editor-in-Chief

PLOS Neglected Tropical Diseases

orcid.org/0000-0003-1765-0002

**Journal Requirements:**

1) We ask that a manuscript source file is provided at Revision. Please upload your manuscript file as a .doc, .docx, .rtf or .tex. If you are providing a .tex file, please upload it under the item type u2018LaTeX Source Fileu2019 and leave your .pdf version as the item type u2018Manuscriptu2019.

2) Your manuscript is missing the following sections: Results, and Methods.  Please ensure all required sections are present and in the correct order. Make sure section heading levels are clearly indicated in the manuscript text, and limit sub-sections to 3 heading levels. An outline of the required sections can be consulted in our submission guidelines here:

https://journals.plos.org/plosntds/s/submission-guidelines#loc-parts-of-a-submission

3) Please insert an Ethics Statement at the beginning of your Methods section, under a subheading 'Ethics Statement'. It must include:

i) The full name(s) of the Institutional Review Board(s) or Ethics Committee(s)

ii) The approval number(s), or a statement that approval was granted by the named board(s)

iii) A statement that formal consent was obtained (must state whether verbal/written) OR the reason consent was not obtained (e.g. anonymity). NOTE: If child participants, the statement must declare that formal consent was obtained from the parent/guardian.].

4) Please upload a copy of Figure Figure 1 which you refer to in your text on page 3. Or, if the figure is no longer to be included as part of the submission please remove all reference to it within the text.

5) Please ensure that all Figure files have corresponding citations and legends within the manuscript. Currently, Figure Figure 2 in your submission file inventory does not have an in-text citation. If the figure is no longer to be included as part of the submission, please remove it from the file inventory.

6) Tables should not be uploaded as individual files. Please remove these files and include the Tables in your manuscript file as editable, cell-based objects. For more information about how to format tables, see our guidelines:

https://journals.plos.org/plosntds/s/tables

7) Some material included in your submission may be copyrighted. According to PLOSu2019s copyright policy, authors who use figures or other material (e.g., graphics, clipart, maps) from another author or copyright holder must demonstrate or obtain permission to publish this material under the Creative Commons Attribution 4.0 International (CC BY 4.0) License used by PLOS journals. Please closely review the details of PLOSu2019s copyright requirements here: PLOS Licenses and Copyright. If you need to request permissions from a copyright holder, you may use PLOS's Copyright Content Permission form.

Please respond directly to this email and provide any known details concerning your material's license terms and permissions required for reuse, even if you have not yet obtained copyright permissions or are unsure of your material's copyright compatibility. Once you have responded and addressed all other outstanding technical requirements, you may resubmit your manuscript within Editorial Manager.

Potential Copyright Issues:

- Please confirm (a) that you are the photographer of Figure 2., or (b) provide written permission from the photographer to publish the photo(s) under our CC BY 4.0 license.

**Reviewers' Comments:**

Reviewer's Responses to Questions

**Key Review Criteria Required for Acceptance?**

As you describe the new analyses required for acceptance, please consider the following:

**Methods**

-Are the objectives of the study clearly articulated with a clear testable hypothesis stated?

-Is the study design appropriate to address the stated objectives?

-Is the population clearly described and appropriate for the hypothesis being tested?

-Is the sample size sufficient to ensure adequate power to address the hypothesis being tested?

-Were correct statistical analysis used to support conclusions?

-Are there concerns about ethical or regulatory requirements being met?

Reviewer #1: The authors present here a case report of paucibacillary leprosy in a 55 year old Florida woman. They highlight many of the classic challenges physicians encounter when diagnosing this infection and their own application of electrophysical techniques to arrive at this diagnosis. The paper is likely to be a benefit to other physicians and serve as a good reminder to consider travel history and possible zoonotic exposure when assessing neuropathic symptoms and skin lesions that do not respond well to ordinary therapies.

Reviewer #2: (No Response)

Reviewer #3: This is a brief case study report of leprosy in a single patient in south Florida.

The objectives of the study are clearly articulated.

Sample size n/a

No statistical analysis.

No ethical or regulatory concerns.

**********

**Results**

-Does the analysis presented match the analysis plan?

-Are the results clearly and completely presented?

-Are the figures (Tables, Images) of sufficient quality for clarity?

Reviewer #1: Yes

Reviewer #2: (No Response)

Reviewer #3: Analysis presented match the analysis plan.

Additional results and an additional Figure may be necessary.

Table results need explanation.

**********

**Conclusions**

-Are the conclusions supported by the data presented?

-Are the limitations of analysis clearly described?

-Do the authors discuss how these data can be helpful to advance our understanding of the topic under study?

-Is public health relevance addressed?

Reviewer #1: Yes

Reviewer #2: (No Response)

Reviewer #3: Additional explanation of the data would benefit the conclusions.

An additional figure of the skin biopsy histology needed.

Authors discuss importance of early diagnosis of leprosy.

Public health relevance is discussed.

**********

**Editorial and Data Presentation Modifications?**

Use this section for editorial suggestions as well as relatively minor modifications of existing data that would enhance clarity. If the only modifications needed are minor and/or editorial, you may wish to recommend “Minor Revision” or “Accept”.

Reviewer #1: Accept

Reviewer #2: (No Response)

Reviewer #3: (No Response)

**********

**Summary and General Comments**

Use this section to provide overall comments, discuss strengths/weaknesses of the study, novelty, significance, general execution and scholarship. You may also include additional comments for the author, including concerns about dual publication, research ethics, or publication ethics. If requesting major revision, please articulate the new experiments that are needed.

Reviewer #1: Minor issues for correction:

Include "travel history to endemic areas and zoonotic exposure" in the abstract and discussion.

Leprosy is an NTD globally, not just in the USA

Based on your assessment this case is ‘autochthonous’, not merely endemic. Much better term.

Armadillos may harbor ‘trillions of leprosy bacilli’ in the entire animal, but only ‘billions of bacilli/per gram"

Reviewer #2: This manuscript, entitled Paucibacillary Leprosy Presenting as Fibular Neuropathy: A Case of Zoonotic Exposure in Endemic Florida by Ruffing et al., reports a case of paucibacillary leprosy diagnosed in Florida, a setting where autochthonous transmission of Mycobacterium leprae has been described. Reporting such cases is important and relevant, particularly to raise awareness among clinicians practicing in regions not traditionally considered endemic. However, despite the potential interest of the case, the manuscript is very poorly written and lacks critical scientific and clinical information, which currently prevents proper evaluation of the diagnosis and the conclusions drawn.

A major concern relates to the confirmation of the diagnosis. The authors state that the patient had “paucibacillary leprosy confirmed by biopsy,” but no details are provided on what was actually done on the tissue. Paucibacillary leprosy is a WHO classification based on the number of lesions and does not, by itself, constitute a diagnostic method. It is therefore essential to specify how leprosy was confirmed in this patient. Was acid-fast bacilli staining (e.g. Fite-Faraco) performed, and if so, what were the results? Was M. leprae PCR carried out on the biopsy? What were the histopathological findings? In addition, it is unclear whether the sample was sent to the National Hansen’s Disease Program (NHDP), which would be standard practice in the United States. Without this information, the diagnosis cannot be adequately assessed.

The manuscript emphasizes zoonotic transmission from armadillos, yet the exposure history provided for the patient remains vague and poorly contextualized. While the authors state that the patient reports frequent soil exposure in her yard, frequent sighting of armadillos, unsuccessful attempts to trap them, and being “knee deep in mud” at times, no temporal or quantitative information is provided. It is unclear how long the patient has lived in Florida, when these environmental exposures began, and for how long she has been exposed to armadillos or potentially contaminated soil. There is also no information on whether exposure preceded symptom onset, which is critical given the long and variable incubation period of leprosy. Simply listing environmental observations without a clear timeline or assessment of exposure intensity does not allow meaningful interpretation and is insufficient to support the strong zoonotic narrative developed in the discussion. This section should either be substantially strengthened with detailed, patient-level exposure data or revised to adopt a more cautious interpretation.

Overall, while the case may be of interest, the manuscript requires major revision. Key diagnostic details are missing, the writing lacks clarity and precision, and the conclusions regarding zoonotic exposure are not adequately supported by the data presented. Substantial improvements would be necessary before this work could be considered for publication.

Reviewer #3: This is a case report of a woman from southern Florida who presented with symptoms of fibular neuropathy who was eventually found with an erythematous rash on her thigh that had central loss of sensation, eventually leading to the diagnosis of leprosy. Up to 20% of armadillos in southern states are infected with M. leprae and this area of central Florida is considered a hot spot with up to 80% of diagnosed cases of leprosy in this state diagnosed in this area. Delayed diagnosis is a problem with leprosy because very few U.S. physicians have any experience with the many symptoms that can occur in the skin and nerves, including neuropathy with loss of sensation within skin lesions as was the case here. The following comments and questions should be addressed.

1. In the Introduction appears “Although rare in the United States, cases have nearly doubled in the past year in Florida according to the Florida Department of Health.” This statement could really be improved by providing some real numbers, for example: “Leprosy in the United States is uncommon with only around 200 cases per year diagnosed in the last ten years, the majority of cases in individuals who have immigrated from endemic foreign countries. Roughly a quarter of leprosy cases are diagnosed in U.S. born individuals who frequently have no known contact with a leprosy patient and are likely to have been infected by an environmental source of M. leprae, most commonly from some contact with armadillos as 10-20% of armadillos in the southern states are infected with M. leprae.” It was stated that the number of leprosy cases in Florida has nearly doubled according to the Florida Department of Health (please cite the web page for this). Please provide the number of cases of leprosy diagnosed in Florida in 2025 for context.

2. The original citation for the occurrence of zoonotic leprosy in the southern U.S. is found in Reference 2 from 2011. The more relevant citation of the cluster of zoonotic leprosy that was found in 15 human cases and 10 armadillo cases that was described in central Florida was more recent and should be included: Sharma R, Singh P, Loughry WJ, Lockhart M, Inman WB, Duthie MS, Pena MT, Marcos LA, Scollard DM, Cole ST, Truman RW. 2015. Zoonotic leprosy in the Southeastern United States. Emerg Inf Dis 21: 2127-2134. doi: 10.3201/eid2112.150501. Please include this citation since the patient described here likely has the same genotype as that found in this cluster, 3I-2-v15.

3. Delayed diagnosis of leprosy in the U.S. is common and can lead to a worsening of nerve damage and disability. There appeared to be a delay in the diagnosis as the patient was initially described as having progressive burning pain in her toes described as possible lumbar radiculopathy followed by developing an erythematous skin plaque that was treated unsuccessfully with topical ointments. How many months exactly passed from the initial symptoms until leprosy was diagnosed, seems like almost 2 years.

4. “Her nerve conduction studies and needle testing are shown (Table 1).” Table 1 does not seem to exist.

5. “Skin biopsy confirmed paucibacillary leprosy.” A figure of the stained (H&E) skin section showing lymphocytic infiltration characteristic of granulomatous disease found with M. leprae would be good to show here. Was there also staining of a section with Fite-Faraco to determine if acid-fast bacilli were present?

6. “Treatment with multidrug therapy is ongoing.” Please be more specific. If this individual was classified with paucibacillary disease, standard treatment with MDT is usually for 6 months with rifampicin, dapsone and clofazimine. If the drug regimen is different, please describe.

7. “armadillos are infected with trillions of bacilli per gram of tissue…” Having knowledge of the absolute numbers of bacilli from armadillo tissues derived from the National Hansen’s Disease Program, it is more likely in the range of between 1E8 to 1E9 (one hundred million to one billion) bacilli per gram of tissue. One trillion bacilli would be 1E12, not possible.

8. The two Tables showing Motor Nerve Results and Sensory Nerve Results show numbers in boxes that are colored green (normal range?) or red (abnormal range?). A summary of the meaning of these results would be helpful in the text. How did these values help or confirm the diagnosis of leprosy? The EMG Table also shows results for right EDB (define abbreviation) with boxes under Ins Act (define abbreviation), Fibs (define abbreviation) and Psw (define abbreviation) with red boxes Inc (increased?), 2+ and 2+, respectively. Please explain what these values indicate and how they led to or confirmed the diagnosis of leprosy.

**********

PLOS authors have the option to publish the peer review history of their article (what does this mean?). If published, this will include your full peer review and any attached files.

If you choose “no”, your identity will remain anonymous but your review may still be made public.

**Do you want your identity to be public for this peer review?**  For information about this choice, including consent withdrawal, please see our Privacy Policy.

Reviewer #1: No

Reviewer #2: No

Reviewer #3: **Yes:**  John S. Spencer

[NOTE: If reviewer comments were submitted as an attachment file, they will be attached to this email and accessible via the submission site. Please log into your account, locate the manuscript record, and check for the action link "View Attachments". If this link does not appear, there are no attachment files.]

**Figure resubmission:**

While revising your submission, we strongly recommend that you use PLOS’s NAAS tool (https://ngplosjournals.pagemajik.ai/artanalysis) to test your figure files. NAAS can convert your figure files to the TIFF file type and meet basic requirements (such as print size, resolution), or provide you with a report on issues that do not meet our requirements and that NAAS cannot fix.

After uploading your figures to PLOS’s NAAS tool - https://ngplosjournals.pagemajik.ai/artanalysis, NAAS will process the files provided and display the results in the "Uploaded Files" section of the page as the processing is complete. If the uploaded figures meet our requirements (or NAAS is able to fix the files to meet our requirements), the figure will be marked as "fixed" above. If NAAS is unable to fix the files, a red "failed" label will appear above. When NAAS has confirmed that the figure files meet our requirements, please download the file via the download option, and include these NAAS processed figure files when submitting your revised manuscript.

**Reproducibility:**

To enhance the reproducibility of your results, we recommend that authors of applicable studies deposit laboratory protocols in protocols.io, where a protocol can be assigned its own identifier (DOI) such that it can be cited independently in the future. Additionally, PLOS ONE offers an option to publish peer-reviewed clinical study protocols. Read more information on sharing protocols at https://plos.org/protocols?utm_medium=editorial-email&utm_source=authorletters&utm_campaign=protocols

# Author comment: Author response to Decision Letter 1

**DOI:** 10.1371/journal.pntd.0014456.r002

29 May 2026

Attachment Submitted filename: Response to Reviewers.docx

# Peer review: Decision Letter 1

**DOI:** 10.1371/journal.pntd.0014456.r003

**Authors:** Mathieu Picardeau, Anil Fastenau, Mathieu Picardeau, Anil Fastenau

10 Jun 2026

Dear Dr. Ruffing,

We are pleased to inform you that your manuscript 'Paucibacillary Leprosy Presenting as Fibular Neuropathy: A Case of Zoonotic Exposure in Endemic Florida' has been provisionally accepted for publication in PLOS Neglected Tropical Diseases.

Before your manuscript can be formally accepted you will need to complete some formatting changes, which you will receive in a follow up email. A member of our team will be in touch with a set of requests.

Please note that your manuscript will not be scheduled for publication until you have made the required changes, so a swift response is appreciated.

IMPORTANT: The editorial review process is now complete. PLOS will only permit corrections to spelling, formatting or significant scientific errors from this point onwards. Requests for major changes, or any which affect the scientific understanding of your work, will cause delays to the publication date of your manuscript.

Should you, your institution's press office or the journal office choose to press release your paper, you will automatically be opted out of early publication. We ask that you notify us now if you or your institution is planning to press release the article. All press must be co-ordinated with PLOS.

Thank you again for supporting Open Access publishing; we are looking forward to publishing your work in PLOS Neglected Tropical Diseases.

Best regards,

Anil Fastenau, M.D., M.Sc.

Guest Editor

PLOS Neglected Tropical Diseases

Mathieu Picardeau

Section Editor

PLOS Neglected Tropical Diseases

Shaden Kamhawi

co-Editor-in-Chief

PLOS Neglected Tropical Diseases

orcid.org/0000-0003-4304-636XX

Paul Brindley

co-Editor-in-Chief

PLOS Neglected Tropical Diseases

orcid.org/0000-0003-1765-0002

***********************************************************

p.p1 {margin: 0.0px 0.0px 0.0px 0.0px; line-height: 16.0px; font: 14.0px Arial; color: #323333; -webkit-text-stroke: #323333}span.s1 {font-kerning: none

Reviewer's Responses to Questions

**Key Review Criteria Required for Acceptance?**

As you describe the new analyses required for acceptance, please consider the following:

**Methods**

-Are the objectives of the study clearly articulated with a clear testable hypothesis stated?

-Is the study design appropriate to address the stated objectives?

-Is the population clearly described and appropriate for the hypothesis being tested?

-Is the sample size sufficient to ensure adequate power to address the hypothesis being tested?

-Were correct statistical analysis used to support conclusions?

-Are there concerns about ethical or regulatory requirements being met?

Reviewer #1: This is a concise caseds report

Reviewer #3: The objectives of this case report are clearly stated.

There is only a single patient case of leprosy in a U.S. born resident from Florida in this report.

No statistical analysis necessary.

No concerns about ethical or regulatory requirements. This is not considered as human research.

**********

**Results**

-Does the analysis presented match the analysis plan?

-Are the results clearly and completely presented?

-Are the figures (Tables, Images) of sufficient quality for clarity?

Reviewer #1: Expert analysis was employed over a complex diagnostic situation.

Reviewer #3: The results have been clarified and revised to be more complete.

Figures and Tables are now presented with explanations.

**********

**Conclusions**

-Are the conclusions supported by the data presented?

-Are the limitations of analysis clearly described?

-Do the authors discuss how these data can be helpful to advance our understanding of the topic under study?

-Is public health relevance addressed?

Reviewer #1: Yes. The authors emphasize the importance of considering leprosy among patients with complex neurological sysmptoms who have a high likelihood of exposure to zoonotic leprosy.

Reviewer #3: Conclusions likely supported by the data presented.

Limitations have been described.

Autochthonous leprosy cases in central Florida have increased likely due to contact with M. leprae shed in the soil from armadillos.

Delay in the diagnosis (in this case it was probably around 18 months from start of symptoms) is a concern as early diagnosis can prevent nerve damage and disability.

**********

**Editorial and Data Presentation Modifications?**

Use this section for editorial suggestions as well as relatively minor modifications of existing data that would enhance clarity. If the only modifications needed are minor and/or editorial, you may wish to recommend “Minor Revision” or “Accept”.

Reviewer #1: Accept.

Reviewer #3: The order of the references should be modified as Reference 5 is the first reference cited in the Introduction. Reference 2 does not seem to appear in the text and Reference 4 appears before Reference 3 in the Discussion.

**********

**Summary and General Comments**

Use this section to provide overall comments, discuss strengths/weaknesses of the study, novelty, significance, general execution and scholarship. You may also include additional comments for the author, including concerns about dual publication, research ethics, or publication ethics. If requesting major revision, please articulate the new experiments that are needed.

Reviewer #1: The authors present here a case report of a 55 year old female patient in Florida with complex neurological symptoms. After an 18 month period involving elaborate efforts to diagnose her condition, they discovered an anesthetic skin lesion consistent with paucibacillary leprosy. In recent years Florida has experienced an increasing number of leprosy cases mainly through zoonotic transmission from armadillos. Further interrogation confirmed that this patient had a high likelihood of exposure to armadillos in her environment, and after initiating standard multiple drug therapy for leprosy the skin lesion and neuropathic symptoms improved. The authors conclude that clinicians should maintain a high index of suspicion for leprosy when neuropathic symptoms coexist with skin lesions among patients with a history of local exposure to armadillos. This is a valuable contribution and should benefit clinicians throughout the Americas.

Reviewer #3: Revisions made have clarified the case report overall.

**********

PLOS authors have the option to publish the peer review history of their article (what does this mean?). If published, this will include your full peer review and any attached files.

If you choose “no”, your identity will remain anonymous but your review may still be made public.

**Do you want your identity to be public for this peer review?**  For information about this choice, including consent withdrawal, please see our Privacy Policy.

Reviewer #1: No

Reviewer #3: **Yes:**  John S. Spencer

# Editor report: Acceptance letter

**DOI:** 10.1371/journal.pntd.0014456.r004

**Authors:** Mathieu Picardeau, Anil Fastenau, Mathieu Picardeau, Anil Fastenau

Dear Dr. Ruffing,

We are delighted to inform you that your manuscript, "Paucibacillary Leprosy Presenting as Fibular Neuropathy: A Case of Autochthonous Zoonotic Exposure in Endemic Florida," has been formally accepted for publication in PLOS Neglected Tropical Diseases.

We have now passed your article onto the PLOS Production Department who will complete the rest of the publication process. All authors will receive a confirmation email upon publication.

The corresponding author will soon be receiving a typeset proof for review, to ensure errors have not been introduced during production. Please review the PDF proof of your manuscript carefully, as this is the last chance to correct any scientific or type-setting errors. Please note that major changes, or those which affect the scientific understanding of the work, will likely cause delays to the publication date of your manuscript. Note: Proofs for Front Matter articles (Editorial, Viewpoint, Symposium, Review, etc...) are generated on a different schedule and may not be made available as quickly.

Soon after your final files are uploaded, the early version of your manuscript will be published online unless you opted out of this process. The date of the early version will be your article's publication date. The final article will be published to the same URL, and all versions of the paper will be accessible to readers.

For Research Articles, you will receive an invoice from PLOS for your publication fee after your manuscript has reached the completed accept phase. If you receive an email requesting payment before acceptance or for any other service, this may be a phishing scheme. Learn how to identify phishing emails and protect your accounts at https://explore.plos.org/phishing.

Thank you again for supporting open-access publishing; we are looking forward to publishing your work in PLOS Neglected Tropical Diseases.

Best regards,

Shaden Kamhawi

co-Editor-in-Chief

PLOS Neglected Tropical Diseases

Paul Brindley

co-Editor-in-Chief

PLOS Neglected Tropical Diseases
